# Estimated undertreatment of carbapenem-resistant Gram-negative bacterial infections in eight low-income and middle-income countries: a modelling study

**DOI:** 10.1016/S1473-3099(25)00108-2

**Published:** 2025-09

**Authors:** Anant Mishra, Rahul Dwivedi, Kim Faure, Daniel J Morgan, Jennifer Cohn

**Affiliations:** a Perelman School of Medicine, Philadelphia, PA, USA; b Global Antibiotic Research and Development Partnership (GARDP), Geneva, Switzerland; c Center for Innovation in Diagnosis, University of Maryland School of Medicine, Baltimore, MD, USA

## Abstract

**Background:**

Carbapenem-resistant Gram-negative (CRGN) bacterial infections are an urgent health threat, especially in low-income and middle-income countries (LMICs), where they are rarely detected and might not be treated appropriately given inadequate health system capacity. To understand this treatment gap, we estimated the total number of CRGN bacterial infections requiring an active agent and the number of individuals potentially initiated on appropriate treatment in eight large LMICs.

**Methods:**

For eight selected countries (Bangladesh, Brazil, Egypt, India, Kenya, Mexico, Pakistan, and South Africa), we estimated deaths associated with CRGN bacterial infections (that were not susceptible to other antibiotics) in 2019 using data from the Global Burden of Disease 2021 study on antimicrobial resistance. We used estimates from the literature to establish infection type-specific case fatality rates and an overall case fatality rate for CRGN bacterial infections. The total number of CRGN bacterial infections requiring an active agent could then be calculated by dividing the total number of CRGN bacterial infection-related deaths by the overall case fatality rate. We estimated the treatment gap (ie, the number of individuals with CRGN bacterial infections who were not appropriately treated) by subtracting from the total number of infections the number of individuals who initiated appropriate treatment, which was estimated using 2019 IQVIA sales data for six antibiotics active against CRGN bacteria, corrected to account for IQVIA's partial data coverage for each country and dose-adjusted by age.

**Findings:**

In 2019, in the eight selected countries, we estimated that there were 1 496 219 CRGN bacterial infections (95% CI 1 365 392–1 627 047) but that only 103 647 treatment courses were procured. The resulting treatment gap (1 392 572 cases [95% CI 1 261 745–1 523 400]) meant that only 6·9% of patients were treated appropriately. The treatment gap persisted even when we used more restrictive assumptions. The most-procured antibiotic was tigecycline (intravenous; 47 531 [45·9%] of 103 647 courses). India procured most of the treatment courses (83 468 [80·5%] courses), with 7·8% of infections treated appropriately (treatment gap 982 848 cases [95% CI 909 291–1 056 405]). The rates of appropriate treatment coverage were highest in Mexico (5634 [5·4%] courses procured; treatment gap 32 141 cases [30 416–33 867]) and Egypt (7572 [7·3%] courses procured; treatment gap 43 258 cases [38 742–47 774]), both with 14·9% of infections treated appropriately.

**Interpretation:**

Infections caused by CRGN bacteria are likely to be significantly undertreated in LMICs. To close this treatment gap, improved access to diagnostics and antibiotics, strengthening of health systems, and research to identify gaps in the treatment pathway are needed.

**Funding:**

Global Antibiotic Research and Development Partnership, supported by the Governments of Canada, Germany, Japan, Monaco, the Netherlands, Switzerland, and the UK, and by the Canton of Geneva, the EU, the Bill & Melinda Gates Foundation, Global Health EDCTP3, GSK, the RIGHT Foundation, the South African Medical Research Council, and Wellcome.

## Introduction

Over the past quarter century, antimicrobial resistance (AMR) has emerged as a major global health threat. It is estimated that almost 1·1 million deaths annually are a direct result of AMR—more than the combined deaths from HIV/AIDS and malaria in 2022.^1^, ^2^, ^3^ Overwhelmingly, low-income and middle-income countries (LMICs) bear this burden—nearly 90% of AMR-related deaths each year are estimated to occur in LMICs.^1^ Today, a child born in Africa is 58 times more likely to die from a drug-resistant infection in the first 5 years of life than one born in a high-income country.^4^ With bacterial drug resistance rates continuing to increase, and without concerted action, almost 40 million cumulative deaths are projected by 2050.^1^

In response to the growing challenge of AMR, global bodies such as the UN General Assembly and G20, alongside national governments (including those of many LMICs), have taken several steps over the past decade to promote stewardship, increase antibiotic access, and foster antibiotic innovation.^5^, ^6^, ^7^ Beyond these necessary steps, accurately identifying infections and administering appropriate antibiotic therapy are fundamental to reducing the mortality associated with AMR. However, to date, there has been little information on how effectively resistant infections are being treated, especially in LMICs. It also remains unclear to what extent barriers along the treatment pathway—from initial health facility presentation to laboratory diagnostic testing or antibiotic access—might drive undertreatment of drug-resistant infections.

**Research in context**

**Evidence before this study**

We searched PubMed and Google Scholar for articles in English, without restrictions based on publication date, up to June 25, 2023, using the search terms (“antimicrobial” OR “antibiotic” OR “Reserve antibiotic”) AND “carbapenem-resistant” AND “access” AND (“developing countries” OR “low- and middle-income countries” OR “Africa” OR “South Asia” OR “Latin America” OR names of countries included in this analysis); we also searched grey literature. Although some perspectives and review articles described treatment barriers or particular instances of antibiotic shortages, no assessment attempted to quantitatively describe the access gap for antibiotics to treat carbapenem-resistant Gram-negative (CRGN) bacterial infections.

**Added value of this study**

This study is the first to quantify a treatment gap for antimicrobial-resistant infections in low-income and middle-income countries (LMICs). Specifically, we used data from the Global Burden of Disease Study's systematic analysis of the burden of bacterial antimicrobial resistance from 1990 to 2021 (also known as the GRAM study) and the health-care database IQVIA to estimate the need for treatment of CRGN bacterial infections and the number of individuals potentially initiated on appropriate treatment in Bangladesh, Brazil, Egypt, India, Kenya, Mexico, Pakistan, and South Africa in 2019. Our findings indicate that, alarmingly, only 6·9% of CRGN bacterial infections in the eight large LMICs analysed were appropriately treated in 2019.

**Implications of all the available evidence**

These results highlight the most recently available picture of the state of care for antimicrobial-resistant infections in the selected LMICs, underscoring the need for meaningful action by global and national policy makers. Given the heterogeneity of the countries selected for analysis, we believe the findings might also apply to other LMICs. Lack of access to appropriate treatment for serious carbapenem-resistant bacterial infections increases morbidity and mortality and potentially increases health-care costs as a result of prolonged hospital stays or other complications. Moreover, undertreatment or inappropriate treatment of these infections further compound the effects of antibiotic resistance. To identify interventions that can improve access to antibiotics for patients with CRGN bacterial infections, more research is needed to better understand and address the barriers to accessing care, timely diagnoses, and appropriate treatment. To organise these efforts, lessons could be drawn from the field of HIV, which has adopted care cascades such as the 95–95–95 targets, resulting in comprehensive coordination across stakeholders and substantial gains in the global HIV response. Analogous comprehensive care cascades for CRGN and other bacterial infections could be developed to guide decision making and track progress, with the opportunity for global stakeholders to set ambitious targets. Finally, the existing access barriers for treatment of antibiotic-resistant infections must be addressed.

To better understand this information gap, we aimed to define and estimate the need for treatment for drug-resistant infections (ie, the total number of resistant bacterial infections) and the number of individuals potentially initiated on appropriate treatment (ie, the two ends of the treatment pathway). Our model focused specifically on the burden and treatment of carbapenem-resistant Gram-negative (CRGN) bacterial infections in eight large LMICs. We chose infections due to CRGN bacteria for our model for two reasons. First, the burden due to CRGN bacterial infections is increasing, with several CRGN pathogens receiving the highest rating (critical) on WHO's priority pathogens list. Second, carbapenems are often the last line of defence, with only a few agents (predominantly WHO Reserve antibiotics) used to treat CRGN infections.^8^, ^9^ Choosing a resistance type with few treatment options rather than one with many (and for which a given treatment can also be used for other resistance patterns) reduces complexity when modelling a treatment pathway. Nonetheless, the method we used to estimate treatment access for CRGN bacterial infections could be refined and applied to the treatment of other antibiotic-resistant infections.

## Methods

### Overview

**Figure 1:**
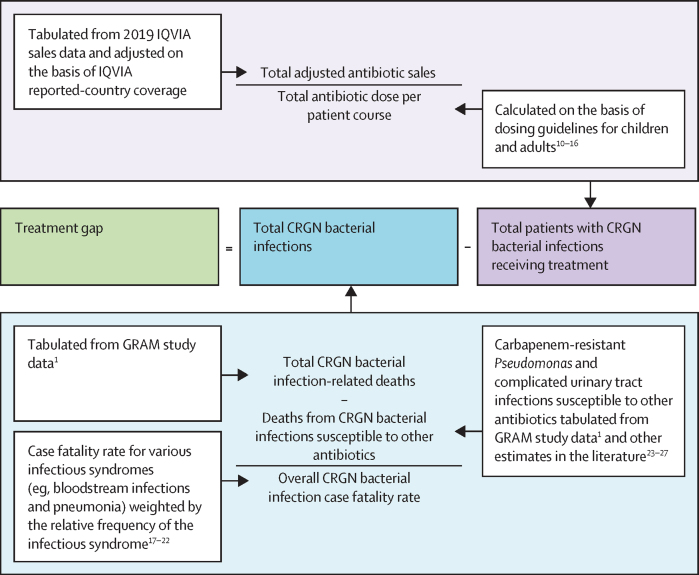
Overview of method to estimate the treatment gap for CRGN bacterial infections CRGN=carbapenem-resistant Gram-negative.

We included eight LMICs: Bangladesh, Brazil, Egypt, India, Kenya, Mexico, Pakistan, and South Africa. These countries were selected for their geographical heterogeneity, substantial burden of antibiotic-resistant infections (based on the literature and on health system characteristics), and existing data availability, particularly related to procurement of antibiotics active against CRGN bacteria. Our methodological approach to estimating the treatment gap for CRGN bacterial infections in these countries is summarised in figure 1 and further details can be found in the appendix (pp 1–5).

### Number of carbapenem-resistant infections (treatment need)

To the best of our knowledge, there are currently no detailed estimates of the total number of CRGN bacterial infections, particularly in LMICs. Thus, we used the following equation: total CRGN bacterial infections = (total CRGN bacterial infection-related deaths – deaths from CRGN bacterial infections susceptible to other antibiotics)/overall CRGN bacterial infection case fatality rate (figure 1).

The number of CRGN bacterial infection-related deaths in 2019 were based on estimates in the Global Burden of Diseases, Injuries, and Risk Factors Study on antimicrobial resistance in 1990–2021^1^ (hereafter referred to as the Global Research on Antimicrobial Resistance [GRAM] study) for the eight countries in our analysis. The specific CRGN bacterial pathogens in the GRAM study were *Acinetobacter baumannii, Citrobacter* spp, *Enterobacter* spp, *Escherichia coli, Klebsiella pneumoniae, Serratia* spp, and *Pseudomonas aeruginosa*.^1^ For these pathogens, the GRAM study provides two estimates of mortality: infection-associated mortality and infection-attributable mortality. Infection-associated mortality is defined as the number of additional deaths compared with a scenario whereby all drug-resistant infections are replaced with no infection, and infection-attributable mortality is the number of additional deaths compared with a scenario whereby all drug-resistant infections are replaced with susceptible infections. Because we were interested in the total number of CRGN bacterial infections, we relied on infection-associated mortality estimates and their associated 95% CIs for our analysis.

We made some adjustments to the total CRGN bacterial infection-related deaths from the GRAM study to ensure that our estimates were as conservative as possible and accurately reflected the deaths of patients from infections that required treatment with an agent with specific activity against CRGN bacterial infections. The first adjustment attempted to account for the fact that, for the selected countries, the literature shows that although resistance remains high, up to 30% of carbapenem-resistant *P aeruginosa* isolates might be sensitive to other antibiotics (eg, fluoroquinolones).^23^ Thus, we conservatively removed 40% of the deaths caused by carbapenem-resistant *P aeruginosa* from the total CRGN bacterial infection-related deaths. For the second adjustment, we excluded a proportion of deaths related to complicated urinary tract infections (UTI), primarily to exclude deaths of patients with infections that could be treated with aminoglycoside monotherapy in line with current guidelines;^24^ data from the literature suggest that up to 40% of complicated UTIs caused by CRGN bacteria in LMICs are susceptible to aminoglycosides.^25^ Thus, for this second adjustment, we assumed that of the 40% of complicated UTIs caused by CRGN bacteria potentially susceptible to aminoglycosides, 85% could have been successfully treated with aminoglycoside monotherapy,^26^, ^27^ and we removed 35% (ie, 40% of 85%) of complicated UTIs caused by CRGN bacteria from further calculations (further details are in the appendix [p 1]).

Next, we estimated an overall case fatality rate for CRGN bacterial infections. We began by pooling available estimates from the literature^17^, ^18^, ^19^ to calculate an overall distribution of CRGN bacterial infection types considered the major drivers of mortality according to the GRAM study^1^ (ie, bloodstream infections, lower respiratory tract infections, intra-abdominal infections, and UTIs). We multiplied these estimates by literature-based case fatality rates for each of these infection types (table 1) to obtain an overall weighted case fatality rate for CRGN bacterial infections.^20^, ^21^, ^22^ Due to the low availability of high-quality data in LMICs, estimates for infection distribution and case fatality rates were drawn from data for adults in high-income countries in North and South America, Europe, and Asia; these infection-specific case fatality rates were drawn from estimated case fatality rates for treated adults identified as having a CRGN infection (predominantly carbapenem-resistant *Klebsiella*). We tested variation in the overall case fatality rate with robustness tests.

**Table 1:** Weighted case fatality rates for carbapenem-resistant bacterial infections, by indication

	**Case fatality rate**	**Contribution to infection burden**	**Case fatality rate (weighted average by indication)**
Lower respiratory tract infections	39%^22^	17%^17^, ^18^	32%
Bloodstream infections	55%^21^	33%^17^, ^18^	32%
Urinary tract infections, intra-abdominal infections, and others	14%^21^	50%^17^, ^18^	32%

### Numbers of individuals initiated on appropriate treatment

To estimate the number of individuals who had started a treatment with activity against a CRGN bacterial infection, we used sales volumes data for 2019 from the IQVIA MIDAS antibiotic sales database for seven of the selected countries and a separate IQVIA audit for Kenya.^28^ The IQVIA MIDAS database captures global pharmaceutical sales of select drugs (both branded and generic) sold directly from manufacturers and wholesalers, as well as sales from hospitals, retail and non-retail pharmacies, and other public and private institutions. For each country, the sales data are collected regularly through surveys and data audits. The total volumes of national sales are projected with the use of an algorithm (developed by IQVIA) that uses regional, sectorial-specific, and distribution channel-specific factors. To further cross-check the data, these sales are annually reconciled for returns and internally validated through a standardised and evidence-based quality assurance programme that accounts for the unique distribution channels of each country. Although not included in the MIDAS database, IQVIA's Kenya data were derived from an audit that similarly captured sales from the aforementioned channels. Overall, across all drugs in all of its included countries, the IQVIA database is estimated to capture more than 85% of sales and has been used extensively to evaluate global medicine use, including use of antibiotics, vaccines, and psychotropic drugs.^29^, ^30^, ^31^

For each country, we extracted detailed sales volumes, if any, for several WHO Reserve antibiotics with activity against CRGN bacteria, including *Enterobacterales*: aztreonam, ceftazidime–avibactam, ceftolozane–tazobactam, colistin, fosfomycin, meropenem–vaborbactam, polymyxin B, and tigecycline (all intravenous medications). Alongside these sales data, IQVIA provided figures for their estimated data coverage for antibiotic sales for each country. To ensure the reliability of our estimates, we focused only on countries with at least 80% reported coverage and explored any variation in IQVIA's data using robustness tests.

For each country–antibiotic combination, we recorded the total volume of sales in grammes or international units (IU) and linearly adjusted these data on the basis of IQVIA's reported data coverage for each country. Next, we used the relevant recommended daily dosing and duration from therapy guidelines for each antibiotic to estimate the total dose of the respective antibiotic a patient would receive during the course of their treatment.^10^, ^11^, ^12^, ^13^, ^14^, ^15^, ^16^ To account for differences in dosing for neonatal and paediatric populations, we adjusted any weight-based dosing on the basis of the GRAM study's distribution of CRGN infection-related deaths in the selected countries:^1^ neonates younger than 28 days, 16·0% of deaths; infants aged 28 days to younger than 5 years, 6·7% of deaths; and individuals aged 5 years and older, 77·3% of deaths. We assumed the mean weight of a neonate to be 3·3 kg, that of an infant aged 28 days to younger than 5 years to be 14·3 kg, and that of an individual aged 5 years and older, including adults, to be 70·0 kg (further details are in the appendix [pp 2–5]).^32^

Finally, by dividing the total adjusted sales volume for each country–antibiotic combination by the total dose required per patient course, we were able to estimate the total number of patients initiated on appropriate treatment (ie, total patients initiated on CRGN bacterial infection treatment = total adjusted CRGN bacterial infection treatment sales [g or IU]/total antibiotic dose per patient course (g or IU; figure 1).

### Robustness tests

To further verify the validity of the estimates for CRGN bacterial infections and appropriately treated patients, we ran two robustness tests. The first tested the estimated total number of CRGN infections by using the lower bound of the 95% CI for the total number of CRGN bacterial infection-related deaths and an overall case fatality rate of 60%. This test was done to estimate the treatment gap with the most restrictive assumptions for estimated infections. Although exact comparisons are difficult, studies of case fatality rates in patients with sepsis in LMICs suggest that the highest rates could be almost double those in high-income countries;^33^, ^34^, ^35^ therefore, we selected 60% as the overall case fatality rate for this test because it is approximately double the rate calculated from high-income country data. The second robustness check tested varying the number of patients treated by adjusting IQVIA's sales coverage for each country. Although IQVIA data and their estimated country coverage are well validated (and we specifically focused on countries with high data coverage), varying the country coverage allowed us a straightforward way to capture any variation introduced from the treatment data.

### Statistical analysis

All descriptive analyses were done with Microsoft Excel version 16.77.

### Role of the funding source

The funders had no role in study design, data collection, data analysis, data interpretation, or the writing of the report.

## Results

Across the eight selected countries, we estimated that there were 478 790 CRGN bacterial infection-related deaths (95% CI 436 925–520 655) and 1 496 219 CRGN bacterial infections (1 365 392–1 627 047) requiring one of the studied WHO Reserve antibiotics in 2019 (table 2). Most of these infections occurred in South Asia, with 1 066 316 (992 759–1 139 873) estimated to occur in India. Kenya and South Africa, the least populated of the countries selected for analysis, had the lowest numbers of CRGN bacterial infections.

**Table 2:** Estimated treatment gap for CRGN bacterial infections in selected countries in 2019

	**Deaths of patients requiring active treatment for CRGN bacterial infections, all ages**^1^**(95% CI)**	**Patients with CRGN bacterial infections (95% CI)**	**Active treatment courses sold for CRGN bacterial infections**	**Estimated treatment gap (95% CI)**
Bangladesh	26 648 (20 821–35 299)	83 276 (65 066–101 487)	802	82 474 (64 264–100 685)
Brazil	32 290 (29 281–35 829)	100 906 (91 502–110 309)	363	100 543 (91 139–109 946)
Egypt	16 266 (14 820–17 711)	50 830 (46 314–55 346)	7572	43 258 (38 742–47 774)
India	341 221 (317 683–364 759)	1 066 316 (992 759–1 139 873)	83 468	982 848 (909 291–1 056 405)
Kenya	5066 (4111–6020)	15 830 (12 848–18 812)	39	15 791 (12 809–18 773)
Mexico	12 088 (11 536–12 640)	37 775 (36 050–39 501)	5634	32 141 (30 416–33 867)
Pakistan	37 641 (31 728–43 555)	117 629 (99 149–136 109)	4124	113 505 (95 025–131 985)
South Africa	7570 (6945–8195)	23 657 (21 704–25 610)	1645	22 012 (20 059–23 965)
Total	478 790 (436 925–520 655)	1 496 219 (1 365 392–1 627 047)	103 647	1 392 572 (1 261 745–1 523 400)

The treatment gap for each country was estimated by dividing the GRAM study's^1^ number of deaths due to CRGN bacterial infections requiring active treatment by the case fatality rate (weighted average; 32%) to estimate the number of patients with CRGN bacterial infections, and then subtracting from this number the number of active treatment courses sold for CRGN bacterial infections, according to IQVIA data. CRGN=carbapenem-resistant Gram-negative.

Despite the large number of infections, we estimated that only 103 647 courses of antibiotics active against CRGN bacterial infections were procured in the study countries in 2019 (table 2), a potential treatment gap of 1 392 572 patients (95% CI 1 261 745–1 523 400) or only 6·9% of patients potentially receiving appropriate treatment. India procured most of the studied antibiotics (83 468 [80·5%] of 103 647 courses), but this amount was only a fraction (7·8%) of its total need (treatment gap 982 848 cases [95% CI 909 291–1 056 405]). Mexico and Egypt had the highest rates of procurement of treatments active against CRGN-active treatment, with each procuring sufficient antibiotics to treat 14·9% of their estimated patients. Bangladesh procured 1·0%, Pakistan procured 3·5%, South Africa procured 7·0%, and India procured 7·8% of the antibiotics needed. Kenya (0·2%) and Brazil (0·4%) had the lowest rates of appropriate treatment procurement.

Overall, across all eight countries and 103 647 courses procured, the two most procured antibiotics were tigecycline (47 531 [45·9%] courses) and colistin (38 356 [37·0%]), whereas ceftazidime–avibactam was the least procured (865 [0·8%]). This trend was mirrored in each individual country, with the exception of Mexico and Pakistan, where fosfomycin and tigecycline were the two most procured antibiotics. Procurement of meropenem–vaborbactam was not recorded by any of the eight countries. India, Brazil, and Mexico each procured at least four different antibiotics, whereas Bangladesh, Kenya, and South Africa procured two or fewer different antibiotics.

We verified the validity of our estimates with robustness tests. First, for the estimate of total CRGN bacterial infections, we used the lower bound of the 95% CI for CRGN bacterial infection-related deaths and an overall case fatality rate of 60%. Even with these more restrictive assumptions, the treatment gap was substantial, with 728 208 infections altogether versus 103 647 treatment courses (ie, 14·2% of patients potentially receiving appropriate treatment). In the second robustness test, we varied the estimate of patients treated by adjusting IQVIA's sales coverage for each country. In the original analysis, the weighted average IQVIA coverage across all countries was 92·0%. Assuming a lower overall sales coverage of 80·0% increased the estimated number of treatment courses from 103 647 to 119 193. Subtracting this number from the 1 496 219 estimated infections in the main analysis resulted in a treatment gap of 1 377 026 courses (ie, 8·0% of patients potentially receiving appropriate treatment), and subtracting it from the 728 208 estimated infections in the first robustness test resulted in a treatment gap of 609 015 courses (ie, 16·4% of patients potentially receiving appropriate treatment).

## Discussion

In this study, we estimated that in eight large LMICs in 2019, 1 496 219 infections were caused by CRGN bacteria requiring treatment with one of the studied antibiotics. Potentially, only 6·9% of these infections were treated appropriately, meaning that almost 1·4 million infections caused by CRGN bacteria were undertreated in these countries. Although most of the infections and treated patients were in south Asia, all eight study countries had substantial gaps in appropriate treatment coverage. The most common antibiotic regimens used were tigecycline and colistin. The treatment gap persisted even with the use of several alternative assumptions, validating the results and highlighting the need for interventions.

To the best of our knowledge, this study provides the first estimates of total infections caused by CRGN bacteria (or any antibiotic-resistant bacteria) and treatment rates in an LMIC setting. To achieve the highest-quality estimates, we selected countries in each global region that were noted to have a high burden of infections caused by CRGN bacteria, as well as those with availability of high-quality data (primarily constrained by the availability of IQVIA data). Comparison with other figures is difficult, given the scarcity of data to inform total numbers of deaths or infections caused by CRGN bacteria beyond the country estimates in the GRAM study.^1^ Nonetheless, the total numbers of bloodstream isolates tracked by South Africa's National Institute for Communicable Diseases AMR database (from both public and private sectors) align with the data from the GRAM study (comparing our estimates with this database showed a difference of approximately 800 infections in 2019).^36^ Similarly, rates of antibiotic consumption across all antibiotic classes in two recent studies were grossly similar to our results—eg, one multinational analysis showed higher rates of antibiotic use in south Asia than in other LMICs, especially those in sub-Saharan Africa, and another recent study focusing on Brazil showed similar but slightly lower antibiotic consumption across all intensive care units in the nation.^29^, ^37^ Despite the large amount of antibiotic sales in south Asia, our study showed appropriate treatment coverage ranging from 1·0% (Bangladesh) to 7·8% (India) in this region. In the other countries in this study, treatment coverage ranged from 0·2% (Kenya) to 14·9% (Mexico and Egypt). Apart from tigecycline and colistin, which were widely used, no other antibiotic was used in more than four countries, highlighting the myriad of country-specific access and clinical factors that shape antibiotic use.

Although our results are only indicative and have limitations, they show that there is almost certainly a substantial gap in the appropriate treatment of CRGN bacterial infections in LMICs. Our results focus on the year 2019, but given the continued rise in CRGN bacterial infections, the effects of the COVID-19 pandemic, and dramatic reductions in global foreign aid, we believe that this gap might have increased. However, preliminary IQVIA data not included in this analysis have shown that some countries have made small but meaningful improvements in the procurement of antibiotics active against CRGN bacteria since 2020 (eg, Brazil). Further monitoring is required to fully track the impact of these global events on the treatment gap. The causes of this gap are likely to be multifactorial, ranging from paucity of access to health-care facilities and inadequate timely diagnostics to barriers to accessing appropriate treatments. Future quantitative and qualitative research will be needed to document the contribution of various barriers to appropriate treatment, but current access barriers should be addressed, including inadequate registration of therapies, high prices, and a scarcity of diagnostics to guide appropriate antibiotic choice.^38^, ^39^

There is currently no concerted, comprehensive approach to address this treatment gap. One approach, similar to strategies used successfully in other fields, could be to deconstruct the treatment pathway for CRGN bacterial infections into a care cascade. Care cascades such as the UNAIDS 95–95–95 (formerly 90–90–90)^40^ targets for HIV (95% of individuals aware of their HIV status, 95% of individuals on antiretroviral treatment, and 95% of individuals virally suppressed) have improved decision making and resource allocation globally. Normative agencies, national governments, and implementing partners worldwide have used the 95–95–95 cascade to identify areas of need, apportion funding, and develop intervention strategies at the global, national, and sub-national levels. The result has been comprehensive coordination across stakeholders and substantial gains in the global HIV response.^41^

**Figure 2:**
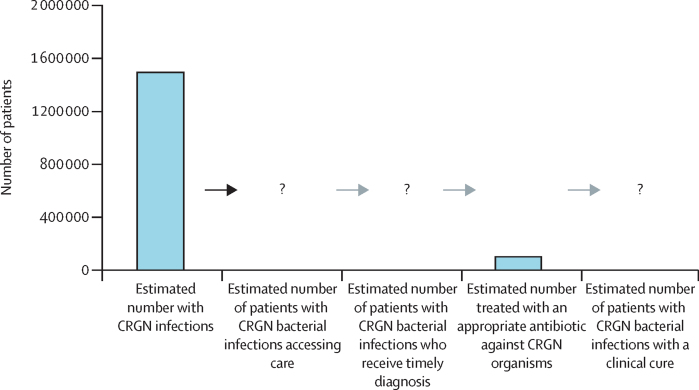
Proposed care cascade for CRGN bacterial infections Bars show estimates from this study for the eight selected countries (Bangladesh, Brazil, Egypt, India, Kenya, Mexico, Pakistan, and South Africa) in 2019. The question marks represent the portions of the cascade for which we do not currently have data. CRGN=carbapenem-resistant Gram-negative.

Given the large-scale success of such care cascades and a need for concerted action, we propose an analogous approach be adopted at the global, regional, and national levels for the treatment of antimicrobial-resistant infections. A simple but comprehensive AMR care cascade would create a powerful tool that all stakeholders could use to target the control and treatment of antibiotic-resistant infections. To this end, building on the cascade endpoints defined in this Article and delineating the intervening steps in the care cascade will be crucial. Figure 2 outlines a proposed cascade that includes our existing data. Tracking the relevant data and updating the cascade could help to highlight the care gaps and aid the design of fit-for-purpose interventions. Other databases of infections and resistance patterns—such as the South African AMR database^36^ and the Indian Council of Medical Research's sentinel sites^23^—could provide additional direct data to inform the cascade; such databases merit increased investment. Real-world data on diagnostic and treatment practices and antibiotic use are also needed.^42^, ^43^, ^44^ The AMR community could set ambitious targets for the percentages of individuals with antimicrobial-resistant infections accessing care, being accurately diagnosed, and receiving appropriate therapy by 2030. Sentinel sites in individual countries and programmes such as WHO's Global Antimicrobial Resistance and Use Surveillance System could build on earlier action plans and track and report on progress towards such targets.^45^ Similarly, care cascades could be constructed and targets set for other bacterial infections.

Our analysis has some limitations; thus, the results must be interpreted with caution. First, estimated need was based on the GRAM study data,^1^ which are modelled data and have inherent limitations. In particular, the GRAM study authors noted that scarcity of microbiological and clinical data in LMICs meant that modelling AMR burden in these settings was challenging, although there have been improvements since the earlier GRAM study.^46^ For these countries, the GRAM study relied more heavily on regional trends and methodological assumptions to inform its calculations than it did for high-income countries, which might have contributed to bias in its estimates. However, we attempted to account for some of this variation using the lower bound of the 95% CI in our first robustness test. We also attempted to correct for complicated UTIs and infections caused by *Pseudomonas* that can be treated with alternative antibiotics. The GRAM study's estimates of carbapenem-resistant *Acinetobacter* infections in south Asia and LMICs were of particular relevance to our analysis. Although these estimates diverged from previous analyses, the authors concluded that the estimates remained consistent with documented rates of carbapenem-resistant *Acinetobacter* infections in these settings. Overall, despite these limitations, the GRAM study is the most comprehensive analysis of bacterial AMR burden to date, reflecting the best and widest range of available data.

Another limitation to our estimates of total CRGN bacterial infections was the relative paucity of LMIC-specific data. Our estimates relied predominantly on syndrome distribution and syndrome-specific case fatality rates in high-income countries, which might have differed from those in LMICs. However, we accounted for this potential limitation by assuming a very high case fatality rate in the first robustness check.

Our estimates of treatment rates for CRGN bacterial infections also have limitations. Antibiotic procurement data were drawn from IQVIA's proprietary database, which is subject to limitations in IQVIA's methodology. Specifically, under-reporting in non-audited market channels or sales in informal markets might not be fully captured. We attempted to account for this possibility in various ways. First, we focused on countries with strong IQVIA data, which generally have a greater number of measurable channels for medicine access, potentially biasing the study towards countries with stronger reporting systems or countries that might have more attractive commercial markets. Additionally, to calculate country-level antibiotic sales, we proportionally adjusted our estimates on the basis of IQVIA's reported coverage of each country to include any uncaptured sales. Inherent to this adjustment was the assumption that the mix of antibiotics in the proportion of sales not covered by IQVIA was similar to that in the sales that were covered. Although we cannot altogether exclude the possibility of differences in the small percentage of sales not accounted for, we believe that any such differences would not substantially affect our results. Moreover, decreasing IQVIA's sales coverage from 92·0% to 80·0% for each country in our second robustness test did not change our conclusions. Another limitation is that IQVIA data relate to sales and might not fully equate to treatment use. Overall, however, these data have been shown to be comprehensive and well validated, with IQVIA's estimates of global antibiotic use remaining the best available.

Limitations that could have led to an overestimation of treatment courses include the assumptions that all of the selected antibiotics were used appropriately and only for the treatment of infections caused by CRGN bacteria and that every patient received only one monotherapeutic treatment regimen. There could have been additional use of other antibiotics not studied (eg, chloramphenicol), but we believe that the use of other antibiotics was minimal and probably insufficient to greatly affect our results. Finally, we assumed that dosing of the antibiotics was similar to that recommended in treatment guidelines for adults and children. Overall, we do not expect any of these limitations to have a meaningful effect on our findings or their interpretation.

In conclusion, we estimated that infections caused by CRGN bacteria were greatly undertreated in several large LMICs in 2019. Inadequate access to appropriate treatment undoubtedly increases morbidity and mortality for patients with CRGN bacterial infections, compounding the effects of antibiotic resistance. Barriers to accessing treatment should be addressed, and more research is needed to elucidate the multiple factors driving disparities. Adoption and further development of a care cascade could support this process and serve as a crucial first step to identify intervention gaps, develop appropriate policies, and improve the targeting of resources to meet the challenge of AMR.

### Contributors

### Data sharing

All data related to calculations of total CRGN bacterial infections will be made available on request. Complete IQVIA sales data are unavailable due to contractual obligations with IQVIA. The corresponding author can be contacted for enquiries.

## Declaration of interests

DJM reports travel support from the Society of Healthcare Epidemiology of America, US Centers for Disease Control and Prevention, WHO, and Infectious Diseases Society of America. All other authors declare no competing interests.
