# Supplementary material for: Estimated undertreatment of carbapenem-resistant Gram-negative bacterial infections in eight low-income and middle-income countries: a modelling study
Source: Lancet Infect Dis. 2025 Sep;25(9):1011–9. doi: 10.1016/S1473-3099(25)00108-2 (PMC12367590; doi:10.1016/S1473-3099(25)00108-2)
Supplement: Supplementary appendix [file mmc1.pdf]

THE LANCET
Infectious Diseases

Supplementary appendix

This appendix formed part of the original submission and has been peer reviewed. We post it as supplied by the authors.

Supplement to: Mishra A, Dwivedi R, Faure K, Morgan DJ, Cohn J. Estimated undertreatment of carbapenem-resistant Gram-negative bacterial infections in eight low-income and middle-income countries: a modelling study. *Lancet Infect Dis* 2025; published online April 30. https://doi.org/10.1016/S1473-3099(25)00108-2.

Supplementary Appendix Table of Contents

1. Supplementary Table 1: Overview of calculations for CRGN deaths/infections 1

2. Supplementary Table 2: Weight-based dosing assumptions 2

3. Supplementary Table 3: Dosing assumptions by drug 3

4. Sample patients treated calculations by drug 3-5

5. References 6

Supplementary Table 1: Overview of data and calculations for determination of total carbapenem-resistant gram-negative (CRGN) deaths and infections/treatment need derived from GRAM study data (The Lancet, 2024)

Country	Total CRGN Deaths[1] [95% CI]	Total CRPA Deaths[1] [95% CI]	CRPA Alternative Treatment[2] (%)	Removed CRPA Deaths [95% CI]	Country Deaths from cUTI[1] (%)	CRGN cUTIs Susceptible to Aminoglycosides[3-6] (%)	CRGN cUTI Deaths Treatable by Aminoglycoside Monotherapy[3-6] (%)	Removed CRGN cUTIs Deaths [95% CI]	Final CRGN Addressable Deaths [95% CI]	CRGN Case Fatality Rate[7-12]	Total CRGN Infections and Treatment Need [95% CI]
Bangladesh	28,918 [22,449, 35,386]	4,405 [3,086, 5,724]	40%	1,762 [1,234, 2,290]	5.1%	40%	86%	507 [394, 621]	26,648 [20,821, 32,476]	32%	83,276 [65,066, 101,487]
Brazil	38,197 [34,496, 41,898]	9,873 [8,618, 11,128]	40%	3,949 [3,447, 4,451]	14.9%	40%	86%	1,958 [1,768, 2,148]	32,290 [29,281, 35,299]	32%	100,906 [91,502, 110,309]
Egypt	18,409 [16,737, 20,081]	4,852 [4,330, 5,374]	40%	1,941 [1,732, 2,150]	3.2%	40%	86%	203 [184, 221]	16,266 [14,820, 17,711]	32%	50,830 [46,314, 55,346]
India	376,460 [349,179, 403,741]	6,8671 [6,0722, 7,6620]	40%	27,468 [24,289, 30,648]	6.0%	40%	86%	7,770 [7,207, 8,333]	341,221 [317,683, 364,759]	32%	1,066,316 [992,759, 1,139,873]
Mexico	15,210 [14,346, 16,074]	6,196 [5,507, 6,884]	40%	2,478 [2,203, 2,754]	12.3%	40%	86%	644 [607, 680]	12,088 [11,536, 12,640]	32%	37,775 [36,050, 39,501]
Pakistan	41,037 [34,456, 47,618]	6,584 [5,222, 7,946]	40%	2,634 [2,089, 3,178]	5.4%	40%	86%	762 [640, 885]	37,641 [31,728, 43,555]	32%	117,629 [99,149, 136,109]
South Africa	8,761 [7,936, 9,585]	2,810 [2,327, 3,293]	40%	1,124 [931, 1,317]	2.2%	40%	86%	66 [60, 73]	7,570 [6,945, 8,195]	32%	23,657 [21,704, 25,610]
Kenya	5,804 [4,673, 6,935]	1,571 [1,183, 1,959]	40%	628 [473, 784]	5.5%	40%	86%	110 [88, 131]	5,066 [4,111, 6,020]	32%	15,830 [12,848, 18,812]
**Total**	**532,796 [484,272, 581,318]**	**104,962 [90,995, 118,928]**	**40%**	**41,984 [36,398, 47,572]**		**40%**	**86%**	**12,020 [10,948, 13,092]**	**478,790 [436,925, 520,655]**	**32%**	**1,496,219 [1,365,392, 1,627,047]**

CRGN (Carbapenem-resistant gram-negative); CRPA (Carbapenem-resistant *Pseudomonas Aeruginosa*); cUTI (complicated urinary tract infection);

Supplementary Table 2: Weight-based dosing assumptions for neonate and pediatric populations based on age distribution of Carbapenem-resistant Gram-negative GRAM study deaths and WHO weight-for-age data.

Age Cohort	GRAM Study Distribution of CRGN Deaths by Age (%)	WHO Average Weight[13] (kg)
Neonate (under 28 days)	16.0%	3.3
Post-neonate to under 5 years	6.7%	14.3
5 years and older	77.3%	70.0

Supplementary Table 3: Dosing assumptions and sample total patients treated calculations by drug for each included antibiotic active against carbapenem-resistant gram-negative (CRGN) infections.

Product	Neonate Dosing	Post-neonate to 5 Years Dosing	5 Years and Older Dosing	Treatment Duration	Total Dose Received by Average Patient Over Treatment Course*	Notes
Tigecycline IV[14]	Not eligible	Not eligible	Loading Dose: 100 mg<br>Maintenance Dose: 50 mg/12 hours	7 days	800 mg	Only given to ages 8 and up
Colistin IV[15,16]	Loading Dose: 134,000 IU/kg<br>Maintenance Dose: 148,000 IU/kg/day	Loading Dose: 134,000 IU/kg<br>Maintenance Dose: 148,000 IU/kg/day	9,000,000 IU/day	10 days	73,791,770 IU	N/A
Polymyxin B IV[17,18]	Loading Dose: 22,500 IU/kg<br>Maintenance Dose: 27,500 IU/kg/day	Loading Dose: 22,500 IU/kg<br>Maintenance Dose: 27,500 IU/kg/day	Loading Dose: 22,500 IU/kg<br>Maintenance Dose: 27,500 IU/kg/day	10 days	16,660,000 IU	N/A
Fosfomycin IV[19]	200 mg/kg/day	300 mg/kg/day	18 g/day	10 days	14.3 g	N/A
Ceftazidime-avibactam[20]	Not eligible	62.5 mg/kg/every 8 hours	7.5 g/day	10 days	71 g	Only given to children aged 3 months and old. Modified distribution in this case results in 92% Ages 5 and older, and 8% post-neonates to age 5.

*This is the total dose received on average over a treatment course when adjusting for each of the three age categories.

Sample Country Tigecycline Calculation:

[Country total adjusted Tigecycline sales in mg] = [Country total Tigecycline sales in mg]/[IQVIA country coverage percentage] = 900,000 mg/90% = 1,000,000 mg

[Country total adjusted Tigecycline sales in mg] / [Total Dose Received by Average Patient Over Treatment Course] = 1,000,000 mg/800 mg = 1,250 patients

Sample Country Colistin IV Calculation:

Average Colistin IV dose/patient course: (Neonate dosing x Neonate Sample %) + (Post-neonate dosing x Post-neonate Sample %) + (Age 5 and older dosing x Age 5 and older Sample %)

Average Colistin IV dose/patient course: (134,000 IU/kg x 3.3 kg +148,000 IU/kg/day x 3.3 kg x 10 days) x 16.0 % + (134,000 IU/kg x 14.3 kg +148,000 IU/kg/day x 14.3 kg x 10 days) x 6.7% + 9,000,000 IU/day x 77.3% = 73,791,770 IU/patient course

[Country total adjusted Colistin sales in IU] = [Country total Colistin sales in IU]/[IQVIA country coverage percentage] = 6,800,000,000 IU/85% = 8,000,000,000 IU

[Country total adjusted Colistin sales in IU]/[Total Dose Received by Average Patient Over Treatment Course] = 8,000,000,000 IU/73,791,770 = 108 patients

Sample Country Polymyxin B IV:

Average patient weight: (Neonate weight x Neonate Sample %) + (Post-neonate weight x Post-neonate Sample %) + (Age 5 and older weight x Age 5 and older Sample %)

Average patient weight: (3.3 kg x 16.0% + 14.3 kg x 6.7% + 70 kg x 77.3%) = 56 kg
Average Polymyxin B IV dose/patient course = Polymyxin dosing x Average patient weight = 22,500 IU/kg * 56 kg + (27,500 IU/kg/day * 56 kg * 10 days) = 16,660,000 IU

[Country total adjusted Polymyxin B IV sales in IU] = [Country total Polymyxin B IV sales in IU]/[IQVIA country coverage percentage] = 4,500,000,000 IU/90% = 5,000,000,000 IU

[Country total adjusted Polymyxin B IV sales in IU] / [Total Dose Received by Average Patient Over Treatment Course] = 5,000,000,000 IU/16,660,000 = 300 patients

Sample Country Fosfomycin IV Calculation:

Average Fosfomycin IV dose/patient course: (Neonate dosing x Neonate Sample %) + (Post-neonate dosing x Post-neonate Sample %) + (Age 5 and older dosing x Age 5 and older Sample %)

Average Fosfomycin IV dose/patient course: (0.2 g/kg/day x 3.3 kg) x 16% x 10 days + (0.3 g/kg/day x 14.3 kg) x 6.7% x 10 days + (18 g/day) x 77.3% x 10 days = 14.3 g/patient course

[Country total adjusted Fosfomycin sales in g] = [Country total Fosfomycin sales in g]/[IQVIA country coverage percentage] = 95,000 g/95% = 100,000 g

[Country total adjusted Fosfomycin sales in g] / [Total Dose Received by Average Patient Over Treatment Course] =100,000 g/14.3 g = 6,993 patients

Sample Country Ceftazidime-avibactam Calculation:

Average Ceftazidime-avibactam dose/patient course: (Post-neonate dosing x Adjusted Post-neonate Sample %) + (Age 5 and older dosing x Adjusted Age 5 and older Sample %)

Average Ceftazidime-avibactam dose/patient course: (0.1875 g/kg/day x 14.3 kg x 10 days x 8.0%) + (7.5 g/day x 10 days x 92.0% = 7.1 g/patient course

[Country total adjusted Ceftazidime-avibactam sales in g] = [Country total Ceftazidime-avibactam sales in g]/[IQVIA country coverage percentage] = 4,500 g/90% = 5,000 g

[Country total adjusted Ceftazidime-avibactam sales in g] / [Total Dose Received by Average Patient Over Treatment Course] = 5,000 g/7.1 g = 704 patients

References

1 Naghavi M, Vollset SE, Ikuta KS, *et al.* Global burden of bacterial antimicrobial resistance 1990–2021: a systematic analysis with forecasts to 2050. *The Lancet* 2024; **404**: 1199–226.

2 2022 Annual Report: Antimicrobial Resistance Research and Surveillance Network. New Delhi, India: Division of Epidemiology and Communicable Diseases, Indian Council of Medical Research https://www.icmr.gov.in/icmrobject/custom_data/pdf/resource-guidelines/AMRSN_Annual_Report_2022.pdf.

3 Tamma PD, Heil EL, Justo JA, Mathers AJ, Satlin MJ, Bonomo RA. Infectious Diseases Society of America 2024 Guidance on the Treatment of Antimicrobial-Resistant Gram-Negative Infections. *Clin Infect Dis* 2024; : ciae403.

4 Mitiku A, Aklilu A, Tsalla T, Woldemariam M, Manilal A, Biru M. Magnitude and antimicrobial susceptibility profiles of Gram-Negative bacterial isolates among patients suspected of urinary tract infections in Arba Minch General Hospital, southern Ethiopia. *PLOS ONE* 2022; **17**: e0279887.

5 Madrazo M, López-Cruz I, Piles L, *et al.* Risk Factors for Bacteremia and Its Clinical Impact on Complicated Community-Acquired Urinary Tract Infection. *Microorganisms* 2023; **11**: 1995.

6 Wagenlehner FM, Gasink LB, McGovern PC, *et al.* Cefepime-Taniborbactam in Complicated Urinary Tract Infection. *N Engl J Med* 2024; **390**: 611–22.

7 Bassetti M, Echols R, Matsunaga Y, *et al.* Efficacy and safety of cefiderocol or best available therapy for the treatment of serious infections caused by carbapenem-resistant Gram-negative bacteria (CREDIBLE-CR): a randomised, open-label, multicentre, pathogen-focused, descriptive, phase 3 trial. *Lancet Infect Dis* 2021; **21**: 226–40.

8 Zilberberg MD, Nathanson BH, Sulham K, Fan W, Shorr AF. Carbapenem resistance, inappropriate empiric treatment and outcomes among patients hospitalized with Enterobacteriaceae urinary tract infection, pneumonia and sepsis. *BMC Infect Dis* 2017; **17**: 279.

9 Soontaros S, Leelakanok N. Association between carbapenem-resistant Enterobacteriaceae and death: A systematic review and meta-analysis. *Am J Infect Control* 2019; **47**: 1200–12.

10 Falcone M, Tiseo G, Antonelli A, *et al.* Clinical Features and Outcomes of Bloodstream Infections Caused by New Delhi Metallo-β-Lactamase–Producing Enterobacterales During a Regional Outbreak. *Open Forum Infect Dis* 2020; **7**: ofaa011.

11 Xu L, Sun X, Ma X. Systematic review and meta-analysis of mortality of patients infected with carbapenem-resistant Klebsiella pneumoniae. *Ann Clin Microbiol Antimicrob* 2017; **16**: 18.

12 Hauck C, Cober E, Richter SS, *et al.* Spectrum of excess mortality due to carbapenem-resistant Klebsiella pneumoniae infections. *Clin Microbiol Infect Off Publ Eur Soc Clin Microbiol Infect Dis* 2016; **22**: 513–9.

13 Weight-for-age | World Health Organization. World Health Organ. https://www.who.int/tools/child-growth-standards/standards/weight-for-age (accessed Sept 24, 2024).

14 TYGACIL (tigecycline) Prescribing Information. Bethesda, MD: Federal Drug Administration, 2013 https://www.accessdata.fda.gov/drugsatfda_docs/label/2013/021821s026s031lbl.pdf (accessed Sept 24, 2024).

15 Colistin Dosing Recommendations and Formulary Guidelines. Univ. Neb. Med. Cent. https://www.unmc.edu/intmed/divisions/id/asp/protected-antimicrobials/colistin.html (accessed Feb 12, 2024).

16 Chibabhai V, Bekker A, Black M, *et al.* Appropriate use of colistin in neonates, infants and children: Interim guidance. *South Afr J Infect Dis* 2023; **38**: 555.

17 Fomicyt powder for infusion. Electron. Med. Compend. https://www.medicines.org.uk/emc/product/100356/smpc (accessed April 9, 2025).

18 Tsuji BT, Pogue JM, Zavascki AP, *et al.* International Consensus Guidelines for the Optimal Use of the Polymyxins: Endorsed by the American College of Clinical Pharmacy (ACCP), European Society of Clinical Microbiology and Infectious Diseases (ESCMID), Infectious Diseases Society of America (IDSA), International Society for Anti-infective Pharmacology (ISAP), Society of Critical Care Medicine (SCCM), and Society of Infectious Diseases Pharmacists (SIDP). *Pharmacotherapy* 2019; **39**: 10–39.

19 Polymyxin B for Injection, Powder, or Solution Prescribing Information. Bethesda, MD: National Institutes of Health https://dailymed.nlm.nih.gov/dailymed/fda/fdaDrugXsl.cfm?setid=b56f18c0-ef5e-4ed9-a5af-f79f3cd189b6&type=display (accessed Sept 24, 2024).

20 Avycaz Prescribing Information. Bethesda, MD: Federal Drug Administration, 2015 https://www.accessdata.fda.gov/drugsatfda_docs/label/2015/206494s000lbl.pdf (accessed Sept 24, 2024).
